# Changes in perspective needed to forge ‘no‐regret’ forest‐based climate change mitigation strategies

**DOI:** 10.1111/gcbb.12921

**Published:** 2022-01-17

**Authors:** Karl‐Heinz Erb, Helmut Haberl, Julia Le Noë, Ulrike Tappeiner, Erich Tasser, Simone Gingrich

**Affiliations:** ^1^ Institute of Social Ecology University of Natural Resources and Life Sciences Vienna Vienna Austria; ^2^ Département de Géosciences École Normale Supérieure Paris France; ^3^ Eurac Research Institute for Alpine Environment Bozen/Bolzano Italy; ^4^ Department of Ecology University of Innsbruck Innsbruck Austria

**Keywords:** carbon sequestration, climate change mitigation, fossil‐fuel substitution, nature‐based solutions, opportunity carbon cost, research strategy

## Abstract

Forest‐based mitigation strategies will play a pivotal role in achieving the rapid and deep net‐emission reductions required to prevent catastrophic climate change. However, large disagreement prevails on how to forge forest‐based mitigation strategies, in particular in regions where forests are currently growing in area and carbon density. Two opposing viewpoints prevail in the current discourse: (1) A widespread viewpoint, specifically in countries in the Global North, favours enhanced wood use, including bioenergy, for substitution of emissions‐intensive products and processes. (2) Others instead focus on the biophysical, resource‐efficiency and time‐response advantages of forest conservation and restoration for carbon sequestration and biodiversity conservation, whilst often not explicitly specifying how much wood extraction can still safeguard these ecological benefits. We here argue for a new perspective in sustainable forest research that aims at forging “no‐regret” forest‐based climate change mitigation strategies. Based on the consideration of forest growth dynamics and the opportunity carbon cost associated with wood use, we suggest that, instead of taking (hypothetical) wood‐for‐fossil substitution as starting point in assessments of carbon implications of wood products and services, analyses should take the potential and desired carbon sequestration of forests as starting point and quantify sustainable yield potentials compatible with those carbon sequestration potentials. Such an approach explicitly addresses the possible benefits provided by forests as carbon sinks, brings research on the permanence and vulnerability of C‐stocks in forests, of substitution effects, as well as explorations of demand‐side strategies to the forefront of research and, in particular, aligns better with the urgency to find viable climate solutions.

## Graphical abstract

Forest‐based mitigation is an important strategy to prevent catastrophic climate change, but large disagreement prevails if it should rely on increased harvest for substituting fossil‐energy based products and processes, or on enhanced carbon sequestration in forest carbon stocks. To move beyond this dichotomy, we argue for a new perspective in sustainable forest research: instead of assessing how maximum wood production can be achieved at low forest emissions, we propose to take a desired forest carbon sink as starting point to quantify compatible harvest levels. Such an approach is suited for safeguarding urgently‐needed carbon sinks and to forge “no‐regret” mitigation strategies.

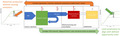

## 1 INTRODUCTION

The world faces a sustainability crisis due to unsustainable patterns of production and consumption. A fundamental societal transformation will be necessary to avoid social and ecological detriments of unprecedented scale (Haberl et al., 2011; Otto et al., 2020; Steffen et al., 2018). Among the many challenges, keeping the global temperature rise below a threshold from 1.5 to 2°K has gained the largest attention and resulted in the formulation of tangible emissions reduction targets (IPCC, 2018; UNFCCC, 2015). Even if ambiguity remains on specific strategies (Rogelj et al., 2021), researchers and an increasing number of political actors agree on the importance of tackling the global climate crisis, whilst safeguarding other elementary social and ecological sustainability dimensions, including e.g. justice and biodiversity.

Prominent modelling exercises illustrate the scope of the challenge, which is massive in terms of the extent of deviation from past trajectories, the volume of emissions to be avoided, as well as in terms of the required speed of action (Creutzig et al., 2021; Roe et al., 2019). Indeed, political targets formulated and agreed upon in the Paris Agreement require that all greenhouse gas (GHG) emissions in 2050 are balanced by sinks and the aggregate net balance is zero (UNFCCC, 2015). To achieve or approximate this ambitious goal, both a minimization of gross emissions and ‘carbon dioxide removal (CDR)’ approaches, e.g. enhancement of terrestrial carbon sinks, will be required. Most scenarios that fit to a 1.5°K target in global model runs rely strongly on land‐based CDR, such as re‐ and afforestation or bioenergy with carbon capture and storage (Roe et al., 2019; BECCS; Turner et al., 2018). Land systems thus face a multidimensional challenge under climate change: They need to continue providing vital resources, such as food, feed, fibres and fuels under a changing climate and for a growing population, whilst at the same time acting as crucially important carbon sinks mitigating further catastrophic climate change (Arneth et al., 2019; Calvin et al., 2009; Olsson et al., 2019).

The management of existing forests is pivotal in this context. Forests play a key role in the global carbon cycle: forest ecosystems alone hold about twice the amount of carbon in the atmosphere pools (Bonan, 2008), and their most dynamic carbon compartment, the biomass stock, is only about two‐thirds of its potential, due to past depletions and recent uses (Erb et al., 2018b). However, whilst agreement prevails in academia that forests, in particular the prevention of deforestation, will play a key role in climate change mitigation, stark disagreement prevails on the concrete forest‐based climate‐change mitigation strategies to be implemented in forests with a long history of intensive management. These forests, mainly situated in the temperate and boreal zones, are characterized by a so‐called forest transition, i.e. the regrowth of forests in terms of area expansion and/or vegetation thickening (Gingrich et al., 2019; Kauppi et al., 2006a; Meyfroidt & Lambin, 2011). For these forests, two contrasting viewpoints can be discerned in the literature: (i) the dominant literature on forest‐based climate solutions suggests enhancing intensive wood use as a renewable source that can replace fossil fuels (‘substitution strategy’), and (ii) more ecologically focused research proposes that ecosystem restoration has great benefits for carbon sequestration in biomass and soils (‘conservation strategy’).

Proponents of the substitution strategy favour enhanced wood to use as an important climate‐change mitigation strategy. Key to this strategy is the acknowledgement of a substitution effect, i.e. a reduction of fossil fuel demand due to the increased use of woody biomass, which eventually allows to compensate for the harvest‐induced forest carbon losses after a so‐called parity time. This parity‐time denotes the time required until a ‘parity point’ is reached, i.e. when the sum of the carbon stock (CS) in forests after harvest plus avoided emissions (substitution) is equal to the CS that would prevail without a harvest (Mitchell et al., 2012; Nabuurs et al., 2017; Ter‐Mikaelian, Colombo, & Chen, 2015; Ter‐Mikaelian, Colombo, Lovekin, et al., 2015). The substitution strategy stresses the income and social benefits of enhanced forest use, as well as the carbon sink effect in socio‐economic CSs such as long‐lived materials and buildings, and the benefit of generating residues that can be used for bioenergy (e.g. Braun et al., 2016; Cambero & Sowlati, 2016; Churkina et al., 2020; Favero et al., 2020; Köhl et al., 2021).

The conservation strategy on the other hand argues with the large carbon sequestration potentials of forests, founded in the gap between actual and potential forest biomass stocks (Erb et al., 2018b; Houghton & Nassikas, 2018; Matuszkiewicz et al., 2021), and additional carbon sequestration in soils (despite the considerable uncertainties related to soils; Bossio et al., 2020; Cook‐Patton et al., 2020). In consequence, this position favours strategies of reduced harvest or exclusion of land use to at least partially exploit these potentials. These approaches stress the advantages of conserving forests in terms of rapid emissions savings and lower monetary costs, as well as high synergies with other targets such as biodiversity conservation (Chazdon & Brancalion, 2019; Griscom et al., 2017; Law et al., 2018; Rockström et al., 2021). In studies adopting this perspective, however, the amount of wood extraction that is compatible with the ecological benefits is usually not quantified.

In our interpretation, this apparently unreconcilable opposition is a direct result of the principal research direction: The substitution strategy takes current resource use and the provision of woody biomass as a substitution for fossil fuel or other GHG‐intensive services as starting points. Studies from this perspective consequently assess—in partial or full carbon accounting approaches (FCA; Marland et al., 2015)—the implication of altered wood uses on the forest carbon‐state and its future dynamics. Sometimes, the effect of wood products substitution for fossil energy is fully considered as a negative flow, which has been fundamentally criticized elsewhere (Harmon, 2019; Leturcq, 2020). Depending on the highly complex mix of assumptions and data, results either show the advantages of substitution over conservation and enhancing the forest carbon‐sink, or vice versa (Brandão et al., 2019; Guest et al., 2013; Macintosh et al., 2015). Overviews and reviews on these issues and different accounting concepts have been published (Mitchell et al., 2012; Ter‐Mikaelian, Colombo, & Chen, 2015).

Beyond these debates, we here point to a conceptual choice made—often implicitly—in these studies. By focussing on the balance of ‘real’ net carbon fluxes following from wood harvest, the ‘opportunity’ carbon costs of wood harvest remain ignored, whilst the substitution benefits are acknowledged. We here challenge this research direction because it is unsuited to tackle the immediate challenges of the climate crisis. Our position is based (1) on the acknowledgement of a systemic opportunity carbon cost of any wood harvest, and (2) on the argument that increasing ecosystem carbon sinks is a more urgent sustainability challenge than maintaining the high levels of resource consumption in countries of the Global North (Rockström et al., 2021). We, therefore, call for a paradigm shift in the exploration of forest‐based mitigation strategies. We argue for taking the preservation or enhancement of carbon sinks, i.e. a pre‐defined target, as starting point for assessments and then exploring sustainable levels of wood supply as derivates of the preserved sink function. This would represent a more tangible and constructive strategy towards the forging of no‐regret forest‐based mitigation options, specifically in light of the urgency of the climate crisis.

## 2 STARTING POINT: FOREST GROWTH DYNAMICS AND HARVEST

Forests are perennial ecosystems, where the majority of individual trees have life spans of several centuries. At the landscape scale and in ecological climax state (i.e. the theoretical final stage of ecological succession), the interplay of natural disturbances and individual tree dynamics results in a relatively stable mosaic of tree‐age distribution. Therefore, in this state, forest CSs tend towards a steady‐state equilibrium (Reichle, 1981; Shugart, 1984). In such old‐grown forests, annual gross carbon fluxes are massive, but the balance of inflows (photosynthesis) and outflows (decay, respiration) tends to oscillate around zero, at least under largely stable climate conditions. In comparison to young and growing forests, the net‐carbon‐flux of old‐grown forests are small. However, old‐grown forests hold a massive CS and prevent organic carbon from being added to the atmosphere (Körner, 2017; Luyssaert et al., 2008). As a consequence, when old‐grown forests are harvested, a fraction of carbon is mobilized from forest biomass and enters socio‐economic pools, whilst another fraction of the biomass (harvest residues) destroyed during harvest remains on site to decay. Wood in socio‐economic pools can have different residence times, ranging from decades, e.g. in construction wood, to less than 1 year, e.g. paper or bioenergy. Eventually, all wood in socio‐economic pools is combusted or decays.

When pristine forests are harvested, the carbon balance is negative, i.e. a net carbon‐emission occurs, even when product stocks are factored in (Harmon et al., 1990; Hudiburg et al., 2019; Keith et al., 2015). The remaining pristine forests, of which currently 49% are located in the tropical South and 42% in the boreal North (*Global Forest Resources Assessment 2020*, 2020), and which contain substantially larger accumulated carbon densities than production forests (Erb et al., 2018a; Keith et al., 2009; Le Noë et al., 2021a; Luyssaert et al., 2008), are currently highly threatened. Conserving these forests is thus of high importance, for the climate as well as for the biodiversity crisis (Goldstein et al., 2020; Potapov et al., 2017).

When wood is harvested not from pristine forests, but already managed forests, the situation is—at first sight—more complex. In such a situation, the initial condition is not a near steady‐state equilibrium of a close‐to‐zero net‐flux, but a dynamic situation of harvest and regrowth, where, at the landscape level, some areas accumulate, whilst others lose carbon. Then, the net‐carbon‐flux is a function of harvest and regrowth, the latter often labelled as ‘increment’. The net carbon outcome depends on the speed of tree growth and the rotation period, i.e. the years between two harvest events at the same plot. In most of the forests in the temperate and large parts of the boreal zone, modern forest management regimes are optimized towards wood production, and forests are harvested at the age of maximum economic profitability, i.e. close to or before the maximum annual increment (Holtsmark, 2012). Owing to the biological growth trajectory of forests, the maximum annual increment is not reached when trees reach maturity, but usually when forests have attained about one half to two‐thirds of their potential CS (Erb et al., 2018b). In production forests, trees are usually harvested every 25–150 years, and only a few trees in a forest composite reach ages beyond that age (Bauhus et al., 2009; Kaipainen et al., 2004), whilst they would be active sinks, i.e. continue to grow and accumulate carbon, for centuries (Luyssaert et al., 2008, 2021; Wutzler & Reichstein, 2007). Wood products, in contrast, usually have much shorter average lifetimes than trees or the difference between cut‐age and maturity, e.g. below 50 years for solid wood and one to a few years for paper (Hudiburg et al., 2019; Keith et al., 2015). Therefore, even when wood harvested from managed forests at a constant rotation period enters long‐lived socio‐economic wood pools, the residence time of carbon in terrestrial carbon pools is, on average, reduced as compared to hypothetical counterfactual no‐harvest scenario (Braun et al., 2016; Erb et al., 2016; Harmon et al., 1990; Hudiburg et al., 2019).

When forests accumulate carbon, as is the case in many regions in the temperate and boreal zone, this can be due to gains in forest area and/or increases in forest carbon density due to vegetation thickening (Kauppi et al., 2006b; Köhl et al., 2015). Whilst forest area expansion is an important driver in specific historical contexts (Le Noë et al., 2020; Meyfroidt et al., 2010; Tasser et al., 2017), forest carbon density increase is currently the more important driver of forest carbon sinks in the temperate and boreal zones (Köhl et al., 2015; Le Noë et al., 2021a). The change in forest carbon density is fundamentally dependent on a specific starting condition: a long history of intensive use or degradation with subsequent recovery, e.g. due to management changes such as species selection or the reduction or abandonment of agricultural practices in forests, recently complemented by changes in environmental conditions, such as nitrogen deposition, carbon dioxide concentration or the length of the growing season. Under these circumstances, both harvest volumes and CSs may increase simultaneously, as has been demonstrated in several cases of temperate forests in the industrialized Global North (Emanuelsson, 2009; Erb et al., 2013; Gimmi et al., 2008; Gingrich et al., 2007; Le Noë et al., 2021b; Magerl et al., 2019; Myllyntaus & Mattila, 2002; Tasser et al., 2007).

A common practice of carbon accounting approaches is to compare the CS of 1 year to the one of the previous year (IPCC, 2006; Keith et al., 2021). If this principle is applied to the above‐described situation, however, it generates results that seem to contradict the principal forest carbon dynamics outlined above: biomass harvest seems to be associated with no net emissions, or, even, with increasing stocks, i.e. net‐absorption. This is, however, a misinterpretation of the fundamental causal relationships between harvest and CSs because stocks grow not as a result of harvest but despite harvests—indeed they result from the recovery process described above (cf. Le Noë et al., 2021a).

## 3 INTRICACIES OF ACCOUNTING: THE OPPORTUNITY CARBON COST OF WOOD HARVEST

The IPCC guidelines on accounting for emissions from forests and other land use on managed land (‘FOLU emissions’) are an integral part of the national account of anthropogenic GHG emissions to the atmosphere (IPCC, 2019). These accounts comprise data on the net carbon flux resulting from land use, which is reflected in the two alternative calculation procedures that are allowed: the gain‐loss approach accounts for all annual in‐ and outfluxes, the stock‐change approach assesses changes in ecosystem CSs. If gains are larger than losses, or stocks increase, carbon dioxide is removed from the atmosphere, and vice versa.

Because the IPCC guidelines represent a tool for monitoring—and not planning—they allow for adding up the independent processes of harvest and regrowth within a forest, and focus on real, verifiable and measurable changes of CSs, i.e. on differences between two points in time. Similarly, but with diverging system boundaries, FCA approaches to account for all fluxes of carbon between the biosphere and the atmosphere, regardless of their origin or causal interrelation (Marland et al., 2015).

The coincidence of a carbon sink with wood harvest in regrowing forests can, based on such accounting schemes, indeed be interpreted as an indication of ‘carbon neutrality’ of extracting wood, even if the carbon it contains is released into the atmosphere in the same year, e.g. through combustion. This is, for instance, a common practice in life cycle assessment‐based studies (see e.g. Giuntoli et al., 2020 for a discussion) and flawed interpretations are not rare, specifically in policy contexts such as the European Renewable Energy Directive (Searchinger, Beringer, et al., 2018). However, this argument presupposes a causal relationship between wood extraction and carbon sequestration in forests that do not exist (see also Birdsey et al., 2018). On the contrary, the effect of forest CS accumulation on forest land remaining forest is eventually the result of two independent processes at the landscape level: (a) on relatively small areas, forests lose carbon because of harvest. (b) On much larger areas, forests recover from such losses and incrementally accumulate CSs, eventually overcompensating the losses on the harvested area (a). Even when forests are—owing to the perennial nature of the plant association—managed in rotation cycles, the processes that occur on the harvested area (a) remain fundamentally independent from the processes that occur on the non‐harvested area (b). If the carbon loss on the harvested area (a) did not occur, carbon‐fluxes on the non‐harvested area (b) would remain the same and the overall carbon sink would be larger.

It is essential in this context to stress that the IPCC accounting guidelines are not intended to deliver information on causal relationships or to inform the design of strategies to reduce net GHG emissions. It definitely makes sense to integrate various sources and sinks of carbon into one account to provide decision‐makers with robust monitoring information. But the convention to mix sources and sinks of various origins to gain an overall picture must not be used to infer causal relationships.

**FIGURE 1:**
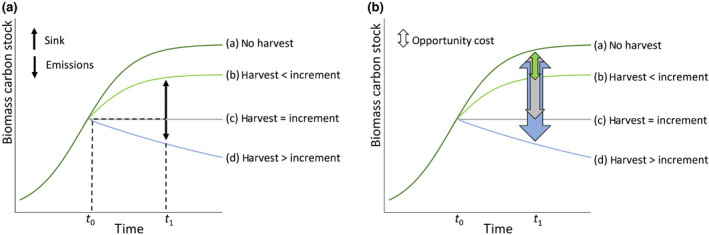
Stylized scheme of carbon stock dynamics in forest biomass at the landscape level following different harvest scenarios, assuming stable forest area. (a) Emissions or sinks are defined as the difference between carbon stocks at two different points in time. (b) Opportunity carbon costs of harvest are defined as the difference between actual carbon stocks and the stock that would prevail without harvest

The systemic, causal interrelation between harvest and CSs in forests can be grasped by comparing CSs of a forest under a specific harvest regime to a no‐use scenario in the hypothetical absence of harvest (corollary, if one is interested in the effect of additional wood demand, comparing the stock dynamics that would occur with and without the activity of regard; (Cowie et al., 2021). Studies are available that provide showcases for the applicability of such approaches (Marques et al., 2019; Searchinger et al., 2018). Such approaches do not quantify the actual carbon emissions or sinks of forest ecosystems, but instead focus on the impact of forest use on the carbon sequestration potential of a forest, the ‘opportunity carbon cost’ or ‘sink foregone’ induced by wood use (Figure 1). The trajectories shown in Figure 1 are well established for living biomass. Evidence on the impact of wood harvest on soil organic carbon (Mayer et al., 2020) suggests—despite the above‐mentioned uncertainties—that including soils in the analysis would not change the general connection between harvest and carbon accumulation, but in fact, increase the carbon impact of harvest on forest ecosystems.

This fundamental principle of opportunity carbon costs of wood harvest is acknowledged in the carbon parity‐time concept (Mitchell et al., 2012; Nabuurs et al., 2017; Ter‐Mikaelian, Colombo, & Chen, 2015; Ter‐Mikaelian, Colombo, Lovekin, et al., 2015). Carbon parity denotes the period it takes until the emissions avoided through wood use plus the carbon accumulated through forest recovery reach the level of CSs that would have manifested in the absence of harvest. The opportunity cost (OC) does not denote a time period, but a hypothetical flux in a given period, which validly applies to all forest uses and at all spatial scales.

Emissions (*E*) or sinks (i.e. removals from the atmosphere or negative emissions) are defined as:

(1) $$E={\text{CS}}_{t1}‐{\text{CS}}_{t0}.$$

Emissions to the atmosphere only occur when the harvest is larger than increment (trajectory d, Figure 1), whilst sinks occur in cases where the harvest is smaller than increment (trajectories a and b). In contrast, the carbon OCs of different use scenarios (b, c and d, Figure 1) are defined as CS in a given year (*t*1) minus CS of the same year under a no‐use trajectory (a):

(2) $$\text{OC}={\text{CS}}_{t1(a)}‐{\text{CS}}_{t1(b,c,d)}.$$

OCs can be assessed when comparing a no harvest counterfactual carbon trajectory (a) with harvest levels that are smaller (b), equal (c) or larger (d) than increment. Accordingly, forests continue growing in CSs (b), stop growing (c) or lose CSs (d) (Figure 1). The OC is the hypothetical difference between (a) and (b) or (a) and (c), and the sum of a physical flux (emission), i.e. the difference between (c) and (d), and the additional hypothetical flux (difference of (a) and (c)).

No‐use counterfactuals quantify the amount of biomass that would grow back to the steady‐state equilibrium of the climax vegetation following a climate‐ and site‐specific growth trajectory and are suited to quantify drivers of actual forest carbon budget trajectories (Gingrich et al., 2021; Le Noë et al., 2021a). Comparing different scenarios to no‐use counterfactuals not only systematically depicts causal interrelationships, it also—indirectly—considers the temporal dynamics of forest change described above, by being sensitive to past depletion in terms of the distance between actual and potential carbon dynamics. Forging and assessing forest‐based mitigation strategies requires the acknowledgement and quantification of these OCs.

It is important here to note that such OCs exist in ‘sustainable’ (Figure 1, cases b and c) as well as in ‘unsustainable’ forestry (case d): The principle of sustainable forestry, formulated centuries ago, holds that harvest volumes are to be kept smaller than increment over larger landscapes (Wiersum, 1995). By definition, this concept does not operationalize the full carbon‐stock implications of wood use—it merely guarantees that carbon gains and losses are equal or gains prevail. Neither the distance from potential biomass stocks, nor the OCs of wood harvest, nor other non‐economic sustainability concerns (e.g. biodiversity or human well‐being) are part of this concept. It remains an open question and key challenge if and to which degree concepts such as ‘climate‐smart forestry’ (Verkerk et al., 2020) or ‘sustainable forest management’ (FAO, 2021) are able to overcome this fundamental trade‐off between harvest, regrowth and carbon‐stock dynamics, underlying the opportunity carbon cost concept.

## 4 THE NEED FOR SUITABLE TOOLS TO WEIGH OPTIONS IN THE FACE OF URGENT FOREST‐BASED CLIMATE‐CHANGE MITIGATION: A SHIFT IN PERSPECTIVE

The urgency of the climate crisis and the need to maximize terrestrial carbon sinks require the identification of no‐regret climate solutions. Currently, the regrowth of forests, mainly in temperate and boreal, i.e. industrialized, regions and transition countries, compensates for about 2.9 Gt C year^−1^ or 30% of anthropogenic emissions each year, so that total global anthropogenic net emissions are 9.4 Gt C year^−1^ instead of 12.3 Gt C year^−1^ (Friedlingstein et al., 2020). Safeguarding this sink is thus a central mandate (Mackey et al., 2013; Rockström et al., 2021). Reducing this sink due to increased harvest—eventually resulting in a forest equilibrium well below the potential, even if apparent carbon neutrality is maintained—will directly translate into pressures to reduce emissions more rapidly in other sectors. But the transformative capacity of many GHG‐intensive sectors is still low and requires time (Tong et al., 2019). We thus argue that we cannot afford to diminish this carbon sink by moving toward a lower forest equilibrium point under high harvest pressure—a claim directly arising from the acknowledgement of the existence of opportunity carbon cost of wood harvest.

**FIGURE 2:**
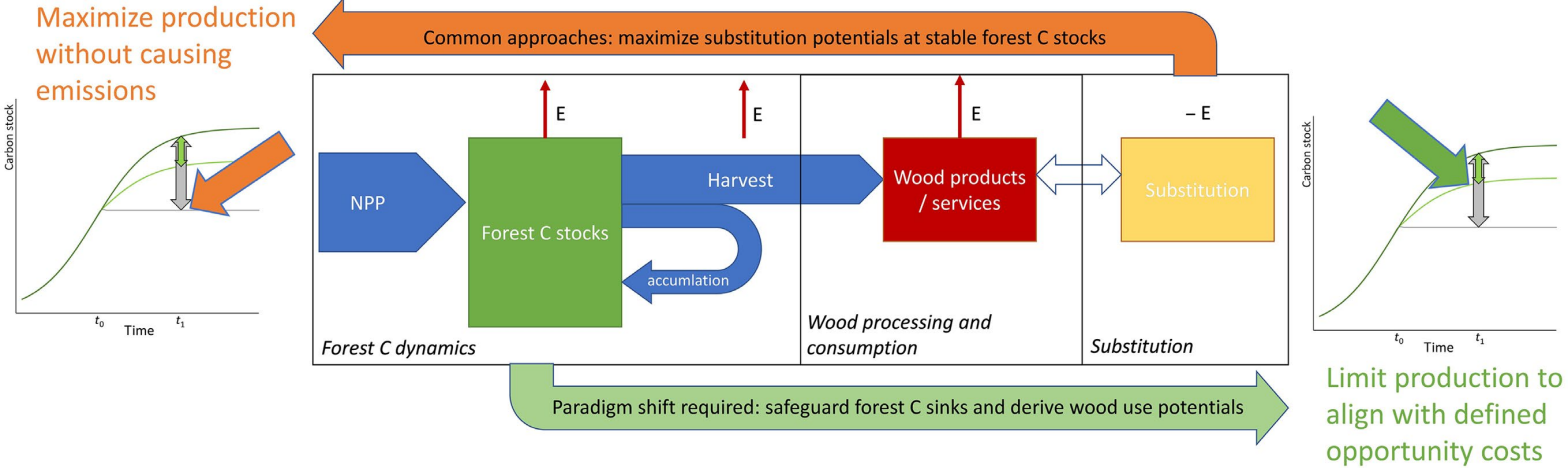
Fundamental research directions of forest‐based climate change mitigation. The currently dominant research direction (substitution strategy) starts with the basic concept of substitution and assesses required wood volumes for these purposes and in consequence the repercussions on forest carbon dynamics (orange arrow on top), with the aim to maximize production whilst avoiding (net) emissions (*E*) from forest stands, forestry operations and processing. The alternative research direction proposed in this text takes the sink preservation as a starting point (or, in other words, a pre‐defined opportunity cost), calculates the ensuing wood harvest potentials and assesses the optimum uses of this resource (green arrow on bottom)

Ignoring OCs of wood use is not adequate in the current situation. Instead, forests need to be viewed as potentially active carbon sinks, and not merely in their role as providers of material and energy. Therefore, we argue for a shift in perspectives when exploring forests' contributions to climate change mitigation: We suggest setting certain levels of forest carbon sequestration as benchmarks and quantify the level of wood use intensity compatible with this aim (Figure 2).

Such a position is fundamentally different from the current mainstream of what sustainable wood use is. Its adoption will result in a paradigm shift in research. This innovative research direction takes the OCs as a ‘benchmark’ of action by setting desired carbon sinks as basic premises and calculating the amount of biomass that can be provided under this constraint. Instead of asking ‘How do we maximize substitution effects of wood whilst keeping land use within a net‐zero‐emission boundary?’, it asks ‘How much wood can we provide whilst preserving a specific sink function of forests?’. In doing so, such an approach is in line with calls for science‐based targets for climate‐change mitigation (Rockström et al., 2021). The size of such a sink target depends on the desired climate pathway and the ability or willingness to reduce emissions in other sectors. From the calculation of this ‘sustainable potential’, research questions on the optimal use, including substitution, of this (then scarce) resource can then follow. Building on scenarios of forest change under a specific expected climate and forest management scheme, a desirable sink strength of forests can be assumed or derived from scenarios that are consistent with 1.5° or 2° global warming targets (e.g. Roe et al., 2019; Rogelj et al., 2021). Then, the wood volumes that can be harvested under this sink strength can be quantified, comprising the different qualities of wood. Eventually, from the different volumes of stem wood and residues provided under these constraints, a number of portfolios of use can be derived, assessed against each other, e.g. in technology‐oriented research, and prioritized, according to societal preferences.

The advantages of such a research direction are obvious: Instead of ignoring sinks or considering as carbon neutral all activities not causing emissions, forests would be explicitly addressed in their function of (potential) carbon sinks. Such a strategy allows one to explore and compare pathways that preserve the currently active carbon sinks and align wood use to future sink potentials, as one of many required strategies to reduce net emissions to the atmosphere. This research strategy is not in opposition to the fundamental idea that forest‐based products would substitute for fossil‐fuel emissions, but it would focus on the prioritization of wood uses and instead of fuelling exaggerated expectations in the performance of land‐based ecosystems in their potential contribution to solving the climate crisis (Mackey et al., 2013).

A key advantage of such a strategy is that it allows one to forge strategies that are based on the precautionary principle. It allows one to factor in the considerable uncertainties related to CSs in forests, as well as their forecasts, e.g. due to future disturbances under expected climate change (Keith et al., 2010; Köhl et al., 2021; Seidl et al., 2014, 2017) that currently hamper the development of optimal carbon management strategies (Valade et al., 2017). Starting with the definition of appropriate carbon sinks allows to choose conservative values on, e.g. forest growth dynamics or sink potentials under climate change, and so warrants the development of no‐regret strategies based on robust knowledge. Research efforts that narrow the prevailing uncertainties will represent key elements of such a research direction.

In addition, the approach is in line with considerations on demand‐side solutions, prioritizing the reduction of resource consumption over preserving current levels of resource use. Whilst demand‐side solutions have been demonstrated to be essential in many aspects of the sustainability debate (Creutzig et al., 2018), surprisingly little research exists with regard to the (reduced) consumption of forest products. At the same time, this approach allows to integrate sustainability perspectives on forests other than CSs and flows in a straightforward manner. The calculation of sustainable carbon potentials can straightforwardly integrate perspectives of other sustainability dimensions, such as biodiversity (Camia et al., 2021) or justice (Scheidel, 2019; Scheidel & Gingrich, 2020).

The urgency of the climate crisis requires proceeding in a balanced manner, based on a full appreciation of cause‐effect relationships and comprehensive treatment of uncertainties regarding all assumptions. Particularly needed are strategies that are valid under the wide range of plausible future scenarios and in the light of the considerable uncertainties. Eventually, many strategies will need to be combined to reach climate targets and avoid detriments of unprecedented scale (IPCC, 2018; Turner et al., 2018). Forest‐based solutions can and need to play a role in this endeavour. But the need for no‐regret climate solutions requires a paradigm shift, which builds upon the time‐buying aspects of preserving and enhancing existing sinks. We argue that this is a more tangible climate‐change mitigation strategy than using forests to merely substitute for other products, specifically in industrialized countries where consumption and emissions are unsustainably high.

## CONFLICT OF INTEREST

The authors declare that they have no conflict of interest.
